# Evolution of Systemic Treatment for Hepatocellular Carcinoma: Changing Treatment Strategies and Concepts

**DOI:** 10.3390/cancers16132387

**Published:** 2024-06-28

**Authors:** Michihisa Moriguchi, Seita Kataoka, Yoshito Itoh

**Affiliations:** Molecular Gastroenterology and Hepatology, Graduate School of Medical Science, Kyoto Prefectural University of Medicine, Kyoto 602-0841, Japan; s1120@koto.kpu-m.ac.jp (S.K.); yitoh@koto.kpu-m.ac.jp (Y.I.)

**Keywords:** hepatocellular carcinoma, systemic therapy, combination of systemic and locoregional therapy, conversion, immunotherapy, molecular targeted agent

## Abstract

**Simple Summary:**

Systemic therapy for hepatocellular carcinoma has been advancing rapidly. With the advent of drugs with high response rates and combination immunotherapy, some patients have attained cancer-free status, while others achieved prolonged survival. Under these circumstances, systemic therapies are being developed not only to address advanced stages but also intermediate and early stages of hepatocellular carcinoma, and treatment concepts and strategies for hepatocellular carcinoma are undergoing substantial changes.

**Abstract:**

Systemic therapy for hepatocellular carcinoma (HCC) has undergone substantial advancements. With the advent of atezolizumab plus bevacizumab (ATZ/BEV) combination therapy, followed by durvalumab plus tremelimumab, the era of immunotherapy for HCC has commenced. The emergence of systemic treatment with high response rates has led to improvements in overall survival while enabling conversion to radical surgical resection in some patients with HCC. In patients with intermediate-stage HCC, new treatment strategies combining systemic treatment and transcatheter arterial chemoembolization (TACE) are under development in clinical trials. Moreover, the addition of local therapies, such as TACE, to systemic treatment according to the treatment effect could achieve a certain percentage of complete response. In the IMbrave050 trial, the efficacy of ATZ/BEV combination therapy was validated in patients predicted to have a high risk of recurrence, especially in those who had undergone radical surgery or radiofrequency ablation for HCC. Therefore, systemic treatment for HCC is entering a new phase for all disease stages. The objective of this review is to organize the current position of systemic therapy for each HCC stage and discuss the development of new treatment methods and strategies, with a focus on regimens incorporating immune checkpoint inhibitors, along with future prospects.

## 1. Introduction

Systemic therapy for hepatocellular carcinoma (HCC) commenced with the introduction of sorafenib [1], which was characterized by a low objective response rate (ORR) and a notable disease control rate (DCR). Consequently, a therapeutic paradigm that focused on adverse event (AE) management and sustained disease control over an extended duration was established. Subsequently, regorafenib [2] emerged as a second-line treatment, which prompted exploration of sequential administration with sorafenib to prolong overall survival (OS), which has become a clinical priority. Lenvatinib [3] was next to enter the arena, marking the first instance of a drug with a non-inferior OS to that of sorafenib, thereby positioning it as a viable first-line treatment. Although superiority in terms of OS was not established, the progression-free survival (PFS) rate of lenvatinib surpassed that of sorafenib. Moreover, considering ORRs of 18.8/40.6% based on RECIST v1.1/mRECIST, the efficacy of lenvatinib was confirmed. Following lenvatinib, ramucirumab [4] and cabozantinib [5] were introduced as second-line treatment options to achieve disease control, given their modest ORRs. Treatment objectives prioritized OS extension by administering sequential therapy while preserving hepatic reserve.

Atezolizumab plus bevacizumab (ATZ/BEV) [6] marked a substantial milestone as a pioneering therapeutic regimen that surpasses sorafenib in terms of OS and PFS. The ORR, as assessed using RECIST v1.1, reached 30%, with complete response (CR) documented in 8% of cases. Moreover, durvalumab plus tremelimumab (DUR/TRE) [7] elicited a superior OS to sorafenib, and long-term follow-up has confirmed cases of long-term survival. Consequently, with systemic treatment, cancer-free status and long-term prognosis have been achieved in some cases. Additionally, durvalumab was found to be non-inferior to sorafenib. Thus, similar to other carcinomas, the era of immunotherapy in the systemic treatment of HCC has commenced.

Advancements in systemic therapy have been prompting shifts in our conceptualization of treatment goals, extending treatment options to patients with intermediate-stage disease and, more recently, demonstrating efficacy in patients with early-stage disease. To enhance treatment outcomes, ongoing research activity is focused on combination therapies incorporating systemic and locoregional therapies.

This review aims to delineate the contemporary landscape of systemic therapy tailored to each stage of HCC, provide an overview of ongoing advancements in treatment modalities, and deliberate on future avenues for treatment development.

## 2. Systemic Therapy

### 2.1. First-Line Treatment

The primary approach for the initial treatment of HCC involves the use of ATZ/BEV or DUR/TRE in situations where combined immunotherapy is feasible. Alternatively, sorafenib, lenvatinib, or durvalumab is recommended when combination immunotherapy presents considerable challenges [8].

As a combination therapy, ATZ/BEV comprises an anti-programmed death ligand 1 (PD-L1) antibody and an anti-vascular endothelial growth factor (VEGF)-A antibody, whereas DUR/TRE constitutes an anti-PD-L1 antibody and an anti-cytotoxic T-lymphocyte-associated protein (CTLA)-4 antibody. Anti-PD-L1 antibodies primarily re-invigorate T cells by binding to PD-L1 on cancer cells during the effector phase of the cancer immune cycle [9,10]. Conversely, anti-CTLA-4 antibodies predominantly function in the priming phase, fostering proliferation and activation of tumor antigen-specific T cells [9,10]. Anti-VEGF-A antibodies not only influence the cancer microimmune environment by diminishing regulatory T cells and tumor-associated macrophages [11], they also hold promise for exerting antitumor effects by inhibiting angiogenesis [12].

As stated earlier, ATZ/BEV represents the first regimen to elicit superior OS and PFS to those of sorafenib, boasting a median OS (mOS) of 19.2 months (95% confidence interval [CI] 17.0–23.7) and a median PFS (mPFS) of 6.9 months (95% CI 5.7–8.6), whereas sorafenib elicited an OS of 13.4 months (95% CI 11.4–16.9) and an mPFS of 4.3 months (95% CI 4.0–5.6), with highly promising outcomes reported in a cohort comprising 80% patients with advanced-stage HCC [13]. Hazard ratios (HRs) were favorable for both OS (0.66; 95% CI 0.52–0.85; descriptive *p* < 0.001) and PFS (0.65; 95% CI 0.53–0.81; descriptive *p* < 0.001). Based on sub-analyses and exploratory studies, ATZ/BEV was reportedly more effective than sorafenib across various tumor stages and in the presence or absence of vascular invasion, rendering it an effective treatment option across diverse patient populations. The ATZ/BEV regimen is characterized by a high ORR and a low progressive disease (PD) rate of 19%. Moreover, the ATZ/BEV regimen has shown a notable capacity for tumor downstaging, facilitating treatment conversion, and fostering cancer-free status [14,15], thereby markedly altering treatment paradigms. Considering the ATZ/BEV regimen, AEs include hypertension, proteinuria, bleeding, thrombosis/embolism associated with bevacizumab, and atezolizumab-induced immune-related AEs (irAEs). Nevertheless, the relatively manageable tolerability profile of ATZ/BEV can substantially maintain the quality of life of patients when compared with sorafenib’s profile [16]. Although findings from the IMbrave150 study, the first clinical trial to verify the superiority of ATZ/BEV over sorafenib in terms of OS and PFS, were reported following an observation period of up to 15.6 months, elucidation of the long-term safety and efficacy in real-world data (RWD) is eagerly anticipated.

Conversely, DUR/TRE represents a groundbreaking combination therapy that employs only immune checkpoint inhibitors (ICIs), constituting the first pharmacotherapeutic regimen for HCC that is devoid of VEGF-inhibitory effects. The DUR/TRE regimen presents a viable option for patients in whom the use of VEGF inhibitors can be challenging, such as those with varices prone to bleeding, refractory hypertension, severe proteinuria, or thrombosis/embolism complications. Hence, this development could pave the way for promising treatment options in the current climate of increasing non-alcoholic steatohepatitis-related HCC [17], frequently complicated by metabolic syndrome represented by hypertension and diabetes, which can lead to chronic kidney or arteriosclerotic diseases that may trigger thromboembolism. Although the PFS of DUR/TRE is comparable to that of sorafenib, and the low DCR of 60.1% raises concerns, a tail plateau, typical of immunotherapy, is suggested in the latter segment of the survival curve, with some instances of prolonged survival observed, offering encouraging prospects. Reportedly, 3- and 4-year survival rates are as high as 30.7 and 25.2%, respectively; if disease control is achieved by evaluation based on RECIST v1.1, prolonged survival can be anticipated (3-year survival rate: 44.6%; 4-year survival rate: 36.2%) [18]. In human trials, one notable issue is the exclusion of patients with Vp4 (extension to main trunk/contralateral branch) from the HIMALAYA trial [7], underscoring the need for clinical trials or RWD to further elucidate the safety and efficacy of the DUR/TRE regimen. In combination therapy with an anti-CTLA-4 antibody and an anti-PD-1 antibody, the onset of irAEs tends to occur earlier than observed when these antibodies are administered individually, and an increase in both the frequency and severity of irAEs has been reported [19], warranting vigilant monitoring. According to the HIMALAYA trial’s results, 20.1% of patients necessitated high-dose steroid therapy (≥40 mg/day) when receiving the DUR/TRE regimen, emphasizing the need for special attention.

The decision to utilize either the ATZ/BEV or DUR/TRE regimen hinges upon the assessment of the risk–benefit equilibrium, considering factors such as tumor status, hepatic reserve, and comorbidities while referring to the findings from the IMbrave150 [6] and HIMALAYA trials. Currently, ATZ/BEV, characterized by its elevated ORR and minimal PD rate, is frequently favored in advanced tumor scenarios. Particularly in instances of pronounced vascular invasion (as demonstrated by the efficacy against Vp4 cases in the IMbrave150 study) and highly aggressive tumors, transitioning to second-line treatment subsequent to progression from first-line therapy may prove challenging. Outcomes of systemic therapy may be predominantly determined by the effectiveness of first-line treatment, thereby underscoring the desirability of a treatment approach that could induce tumor shrinkage and maintain a low PD rate as a viable option. Conversely, for patients with advanced-stage HCC with relatively low intrahepatic tumor burden and relatively less aggressive status, DUR/TRE may be an option owing to the relatively high probability of 3- or 4-year survival after disease control is achieved.

Llovet et al. [20] and Bejjani et al. [21] have proposed a decision-making framework for treatment options based on considerations for administering bevacizumab. The selection is contingent upon the assessment of risk factors for gastrointestinal bleeding, such as the presence of varices. Notably, ATZ/BEV is preferred when the risk is low, whereas DUR/TRE is favored when the risk is deemed high. This approach appears to be frequently employed in actual clinical practice. However, it is anticipated that the presence or absence of other comorbid conditions, including intractable hypertension, proteinuria, thrombosis/embolism, and diminished cardiac function, would also impact the decision-making process in terms of bevacizumab administration. Notably, in the IMbrave150 study, the use of anti-platelet agents/anticoagulants was an exclusion criterion for enrollment, except for certain anti-platelet agents. Nonetheless, in real-world clinical settings, numerous patients rely on these medications owing to the presence of underlying conditions, thereby highlighting the pros and cons of administering bevacizumab—a pertinent issue. Nevertheless, in cases where varices posing a high risk of bleeding are absent, and thrombosis/embolism precipitating the use of anti-platelet drugs or anticoagulants is clinically stable, retrospective studies have indicated that bevacizumab can generally be employed without considerable issues, even in conjunction with anti-platelet drugs/anticoagulants [22]. It is anticipated that the accumulation of additional cases involving ATZ/BEV and DUR/TRE will provide further insights, clarifying the optimal utilization of these treatment modalities. Moreover, it is imperative to identify and establish a biomarker to differentiate between the two regimens.

Conversely, when immunotherapy is contraindicated owing to complications (e.g., highly active autoimmune disease), molecularly targeted agents (MTAs), such as sorafenib or lenvatinib, are considered to be viable options. On the other hand, even if the introduction of ICIs is deemed feasible, concerns regarding the initiation of combination immunotherapy, such as ATZ/BEV or DUR/TRE, may arise owing to issues such as the presence of comorbidities. In such scenarios, durvalumab could be recommended as an alternative. This decision may stem from concerns that concurrent administration of tremelimumab could escalate the frequency and severity of irAEs. Durvalumab may be preferred in cases where the reluctance to co-administer tremelimumab or bevacizumab can be attributed to patient characteristics or the presence of comorbidities.

Conversely, there are also scattered reports of RWD on ATZ/BEV therapy showing that the efficacy is somewhat poor in patients with HCC with non-HBV non-HCV (NBNC) background liver disease [23,24]. Therefore, there may be cases where MTA is chosen instead of combined immunotherapy, depending on the etiology, even if the introduction of combined immunotherapy is not difficult. Additionally, both basic research and meta-analyses using clinical trial results suggest that immunotherapy may be less effective for non-alcoholic fatty liver disease (NAFLD)-associated hepatocellular carcinoma than for virus-associated hepatocellular carcinoma [25]. However, an IMbrave150 post hoc analysis reported no differences in ORR, PFS, or OS by etiology [26]. Since the meta-analysis of clinical trials included results from the KEYNOTE-240 [27] and CheckMate459 [28] trials, in which patients were treated with anti-PD-1 antibody monotherapy, and since there may be various biases in the retrospective studies, at this point, it is difficult to say that there are differences in the efficacy of ATZ/BEV based on etiology. Thus, it is difficult to say at this point that there is clear evidence that differences in etiology dictate the choice of therapeutic agents. Further accumulation of clinical data and basic studies will be necessary in the future.

When considering the choice between sorafenib and lenvatinib, lenvatinib is frequently preferred, because the REFLECT trial demonstrated its favorable ORR and PFS despite the lack of OS superiority, and meta-analysis also showed similar results [29]. Regarding the utilization of durvalumab monotherapy versus MTAs, lenvatinib has elicited promising ORR and PFS outcomes, while durvalumab monotherapy exhibits a favorable duration of response. Given that responder cases are anticipated to experience relatively prolonged survival, it is prudent to determine the treatment approach by weighing the risks associated with age, liver function, and comorbidities, as well as by assessing the risk–benefit balance between MTAs and durvalumab monotherapy. Currently, there is a lack of evidence supporting systemic therapy for Child–Pugh (C-P) B cases, representing an unmet medical need. Nevertheless, considering the outcomes of nivolumab therapy in C-P B cases [30], durvalumab could be safe and efficacious for treating patients with C-P B. Conversely, as reported by Kobayashi et al., patients with C-P B may fail to tolerate lenvatinib owing to elevated blood levels [31].

Additionally, the development of new first-line treatments has been ongoing (Table 1)—for example, the COSMIC-312 and LEAP-002 trials, whose results have been published. In the COSMIC-312 study [32], cabozantinib + atezolizumab and sorafenib elicited mPFS rates of 6.8 months (99% CI 5.6–8.3) and 4.2 months (2.8–7.0), respectively, with an HR of 0.63 (99% CI 0.44–0.91, *p* = 0.0012). Despite the significantly substantial extension of PFS, there was no significant improvement in terms of OS: cabozantinib + atezolizumab and sorafenib showed mOS of 15.4 months (96% CI 13.7–17.7) and 15.5 months (12.1–not estimable), respectively, with an HR of 0.90 (96% CI 0.69–1.18; *p* = 0.44). In the LEAP-002 study [33], lenvatinib + pembrolizumab and lenvatinib therapy elicited mOS of 21.2 months (95% CI: 19.0–23.6) and 19.0 months (95% CI: 17.2–21.7), respectively (HR 0.84; 95% CI 0.71–1.00; stratified log-rank *p* = 0.023). Based on RECIST v1.1, lenvatinib + pembrolizumab and lenvatinib were associated with mPFS of 8.2 months (95% CI: 6.4–8.4) and 8.0 months (95% CI: 6.3–8.2), respectively, HR 0.87 (95% CI 0.73–1.02; stratified log-rank *p* = 0.047). Although both results were statistically significant (*p* < 0.05), the preset *p*-value could not be reached, resulting in a negative trial.

The objective of the CheckMate 9DW trial (NCT04039607), an ongoing phase 3 trial in advanced HCC, is to assess the OS superiority of nivolumab plus ipilimumab when compared with sorafenib or lenvatinib monotherapy. Results were presented at ASCO 2024 and showed MST of 23.7 months for nivolumab plus ipilimumab vs. 20.6 months for lenvatinib or sorafenib (HR, 0.79; 95% CI, 0.65–0.96; *p* = 0.0180), showing the OS superiority of nivolumab plus ipilimumab over lenvatinib or sorafenib. The response rate was also significantly better for nivolumab plus ipilimumab than for lenvatinib or sorafenib (36% vs. 13%; *p* < 0.0001). In addition, the study reported a median DOR of 30.4 months for nivolumab plus ipilimumab compared to 12.9 months for lenvatinib or sorafenib [34]. This could critically impact future first-line treatment choices and treatment concepts. More detailed reports are awaited. Meanwhile, a phase 3 study (IMbrave152 trial; NCT05904886) has been initiated to assess the efficacy and safety of tiragolumab when administered in combination with ATZ/BEV. Furthermore, considering cases with performance status (PS) 0 or PS1 and C-P 7 or 8 points, those with Child–Pugh (C-P) A and ECOG performance status (PS) 2, and those with C-P A and PS0 or PS1 with a tumor thrombus in the main trunk of the portal vein, the phase 3 SIERRA trial (NCT05883644) is underway to evaluate the incidence of potential treatment-related grade 3 or 4 AEs and the response rate of DUR/TRE. Systemic therapy for C-P B or PS2 represents an unmet medical need, and results from these trials are eagerly anticipated.

Table 2 shows the percentage of Gr 3 or higher adverse events for each drug and regimen reported in pivotal trials that occurred in 3% or more of all patients, along with the percentage of adverse event discontinuations. For regimens using immune checkpoint inhibitors, the percentage of steroid use for immune-related/immune-mediated adverse events is also shown, while for DUR/TRE and DUR, the percentage of high-dose steroids used is shown. This information is very useful and informative when selecting drugs.

### 2.2. Second-Line Treatment

Considering the use of MTAs, it is crucial to connect not only the first-line treatment but also the second-line and subsequent treatments sequentially. In immunotherapy for HCC, the crucial impact of post-treatment on OS has been reported. Treatment must be conducted while persistently paying attention to liver function and PS, which are the optimal factors for systemic therapy.

The mOS/mPFS of ATZ/BEV in the IMbrave150 trial and those of DUR/TRE in the HIMALAYA trial were 19.2/6.9 and 16.43/3.78 months, respectively, which resulted in ~12-month survival after progression on first-line treatment in both trials. These findings underscore the significance of PPS in OS. Additionally, the rate of treatment transition after ATZ/BEV was approximately 50%, indicating a relatively high transition rate [35]. The DUR/TRE regimen has been in clinical use for approximately one year, and forthcoming reports and insights from ongoing studies are eagerly anticipated.

If ATZ/BEV or DUR/TRE is employed as the first-line treatment, there is currently no systemic therapy with an evidence level that supports use as a second-line treatment. Treatment selection will be determined with reference to pivotal trial data and RWD, considering factors such as the patient’s condition at the completion of primary therapy (including tumor status, liver function, performance status, and effects of adverse events), comorbidities, and other relevant considerations.

In a retrospective study, Persano et al. reported that lenvatinib, selected from among various MTAs as a second-line therapy after ATZ/BEV, demonstrated the best OS [36]. Similarly, Hiraoka et al. performed a retrospective study and found that lenvatinib after ATZ/BEV exhibited comparable safety and efficacy to first-line therapy [37]. Future studies collecting additional cases could provide further insights. Moreover, the potential of introducing a treatment regimen where DUR/TRE follows ATZ/BEV therapy could be considered. In numerous instances, the use of VEGF-based drugs as second-line treatment can be challenging owing to bevacizumab-induced AEs. However, DUR/TRE may offer an alternative in such scenarios. Although there are some reports regarding the administration of consecutive ICI therapy, a consensus has yet to be reached. Clarification regarding the efficacy and safety of combined immunotherapy after combined immunotherapy, as well as determination of the optimal sequence (ATZ/BEV → DUR/TRE or DUR/TRE → ATZ/BEV), is eagerly awaited. In the future, DUR/TRE may be introduced as a subsequent line of therapy. However, in such cases, the anti-VEGF effect of the previous treatment may be forfeited. To date, no treatment scheme capable of “switching off” VEGF inhibition during sequencing therapy for HCC has been established. Consequently, it is also imperative to ascertain whether discontinuing the VEGF-inhibitory effect may lead to rapid deterioration. Furthermore, there are currently no reports on second-line treatment for cases where DUR/TRE was initially introduced as first-line therapy. Future investigations need to clarify this aspect through RWD.

When an MTA is selected as the first-line treatment, the approach differs depending on the use of lenvatinib or sorafenib. In cases where lenvatinib is employed as first-line therapy, evidence for second-line therapy or beyond is currently lacking; therefore, there is no established preferred drug, and a subsequent drug will be selected from among several drugs depending on the individual case. Therefore, treatment selection should be guided by pivotal studies and RWD, considering factors such as the patient’s status at the completion of primary treatment (for example, tumor-related factors, liver function, performance status, and effects of adverse events) and comorbidities, among other considerations. In contrast, if sorafenib is selected as first-line therapy, the development of second-line therapy typically operates under the assumption that sorafenib served as the prior treatment. Thus, there is evidence supporting the use of regorafenib, ramucirumab, and cabozantinib, and the selection of each drug is based on their distinct characteristics. Depending on the underlying reason for not selecting combination immunotherapy at the time of choosing first-line treatment, there may be instances where combination immunotherapy is selected as second-line therapy. In scenarios where durvalumab monotherapy is selected as the first-line treatment, the treatment approach follows a similar trajectory to the selection of lenvatinib monotherapy.

Regarding treatment advancement, a phase 3 study (IMbrave251 study; NCT04770896) focusing on cases that progressed with ATZ/BEV is currently underway. The objective of this trial is to compare OS rates between patients who switched to sorafenib or lenvatinib and those who added sorafenib or lenvatinib to atezolizumab.

### 2.3. Switching from First-Line to Second-Line Treatment

The decision to switch from first-line to second-line therapy is typically based on PD or intolerance to the first-line treatment. As previously mentioned, sequential systemic therapy, whether involving combined immunotherapy or MTAs, could potentially improve prognosis. It is crucial to connect treatments sequentially in a timely manner while maintaining C-P A and PS 0 or 1, which are factors for the induction of pharmacotherapy and for maximizing the effect of each treatment. However, in the IMbrave150 study, among the 236 ATZ/BEV-treated patients evaluated for PD according to the RECIST criteria, 132 continued to receive ATZ/BEV for a period [38]. Remarkably, the OS in this subgroup was 24.1 months [38]. Although there exists a bias in favor of the perceived clinical utility of ATZ/BEV, in situations where evidence-based treatment options subsequent to ATZ/BEV are lacking, it is necessary to carefully consider the circumstances at the time of PD. Continuing ATZ/BEV for a certain duration may be a viable option in some cases. Nevertheless, it should be noted that these cases may encompass instances of pseudo-progression [39], and caution is warranted regarding imaging changes that are specific to immunotherapy. When utilizing ICIs, the use of irRECIST [40] rather than RECIST may be advantageous. Conversely, when administering immunotherapy, instances of hyperprogression [41,42] have been documented, albeit with relatively low frequency. Thus, imaging evaluation may be difficult in immunotherapy, and tumor markers could be useful indicators in such cases. Fluctuations in tumor markers during the early stage of treatment induction were found to be closely associated with subsequent imaging evaluation and outcomes such as PFS in ATZ/BEV therapy [43,44], as observed with MTA therapies such as lenvatinib [45]. Regarding the timing of transitioning to second-line therapy, it is important to consider the PD decision based on RECIST evaluation, along with the tumor status, mode of progression, changes in tumor markers, ECOG PS, liver function, and occurrence of adverse events. The use of tumor markers may be essential in the early stage of treatment induction to determine whether or not imaging evaluation should be performed earlier to avoid missing the timing of the next treatment transition.

### 2.4. Intermediate Stage

Systemic therapy is the standard treatment in advanced-stage HCC, and treatment should typically proceed as described above for the first-line, second-line, and subsequent lines of treatment. Conversely, in cases of intermediate-stage HCC, locoregional treatment options, such as transcatheter arterial chemoembolization (TACE) and, in some situations, radiofrequency ablation (RFA), may be applicable. These methods, unique to HCC and distinct from treatments for other cancers, could offer better outcomes. Thus, in the intermediate stage, systemic therapy is typically indicated for cases in which locoregional therapy is ineffective or not indicated. Therefore, it is essential to comprehensively clarify the advantages, challenges, and limitations of both systemic therapy and locoregional therapy, enabling informed treatment selection and strategy development.

TACE has historically served as the standard treatment for intermediate stages of HCC [8]. However, with the introduction of systemic therapy, the notion of TACE refractoriness has arisen. Furthermore, as systemic therapy continues to advance, the concept of TACE unsuitability has emerged and gained prominence in recent years.

TACE refractoriness is defined as a state where TACE is ineffective for two consecutive treatments, or when vascular invasion/extrahepatic metastasis occurs [46]. Patients who are unresponsive to TACE may achieve a better prognosis by transitioning to systemic therapy, such as sorafenib, rather than continuing with TACE. However, retrospective analyses have indicated that approximately 20–30% of patients identified as TACE-refractory have already progressed to C-P B status [47]; as a result, some patients may not benefit from systemic therapy. In light of these findings, Kudo et al. employed propensity score matching to compare outcomes between cases initiating treatment with lenvatinib and those initiated with TACE [48]. The retrospective study revealed significantly improved PFS and OS in the lenvatinib-initiated group. Although the study relied on two small cohorts from different historical contexts, introducing various biases that warrant consideration, this report also served as one of the backgrounds, and the concept of introducing systemic therapy prior to TACE, known as TACE unsuitability, has been proposed [47]. Within this framework, the up-to-seven criteria, which is one parameter assessing tumor burden, was proposed as an indicator of TACE unsuitability. However, not only the up-to-seven criteria but various tumor burden statuses, such as the up-to-eleven criteria [49] or the presence of eight or more nodules [50], along with a maximum tumor diameter of ≥5 cm in cases with 4 to 7 nodules [50], have been reported to potentially influence the selection of TACE and systemic therapy. Based on retrospective analyses, tumor burden plays a pivotal role in determining the efficacy of TACE in intermediate stages. Moreover, recent studies indicate that tumor localization [51] and radiological features of the tumor can impact TACE’s efficacy [52]. In clinical practice, the choice between TACE and systemic therapy is guided by a comprehensive evaluation of various factors, such as tumor-related factors, liver reserve capacity, the efficacy of systemic therapy, PS, and patient comorbidities.

Lenvatinib reportedly elicits marked efficacy, particularly in intermediate stages of HCC, and unlike TACE, its effectiveness remains consistent regardless of the radiological features of the tumor [53]. This may substantially enhance the prognosis following the implementation of systemic therapy. However, findings from a retrospective study conducted by Kobayashi et al. suggest that although the prognosis of patients with advanced-stage HCC has significantly improved over time with the introduction of MTAs, the prognosis of patients with intermediate-stage disease has seen a comparatively modest improvement [54]. Patients with intermediate-stage disease constitute a highly heterogeneous group, which complicates the uniform assessment of prognosis. Nonetheless, the endeavor to enhance prognosis in the intermediate stages underscores the importance of leveraging more potent systemic therapy and exploring combination approaches with other treatments.

Table 3 summarizes the representative prospective studies. The REPLACEMENT study is a phase 2 trial investigating the introduction of ATZ/BEV in patients with tumor burden beyond the up-to-seven criteria in intermediate-stage HCC who have not undergone TACE or systemic therapy. The initial findings, presented at ILCA2023, demonstrated a response rate of 45.9% (95% CI: 34.3–57.9) according to mRECIST evaluation, along with a PFS of 9.1 months (95% CI: 7.1–10.2), thereby offering promising outcomes [55]. Another objective of the REPLACEMENT study is to conduct an exploratory comparative analysis with a separately collected TACE cohort, with further analyses (including OS) anticipated for publication. Although such comparative analyses across different studies inherently introduce selection bias, thereby limiting the attainment of high-level evidence, they represent a valuable effort in addressing the clinical question regarding the sequencing of ATZ/BEV or TACE as the initial treatment modality. Considering these limitations, efforts are underway to develop a randomized phase 3 trial, the ABC-HCC study (NCT04803994), as a global endeavor aimed at addressing this clinical question. The ABC-HCC study will provide valuable insights into the optimal treatment approach for patients with intermediate-stage HCC beyond up-to-seven criteria.

Since TACE is a locoregional therapy, differences in treatment results have been reported based on tumor burden [50,56] and localization site [51]. Moreover, the possibility of reduced efficacy has been reported in the non-simple nodular type based on the gross morphology on pretreatment imaging [52,56], as well as in the heterogeneous contrast-enhancing form of tumors [57]. In contrast, regarding ATZ/BEV treatment, generally, a good response was reported even for the non-simple nodular type based on gross tumor morphology, which is more likely to be aggressive [58]. A sub-analysis of IMbrave150 showed no difference in antitumor efficacy based on tumor size [59]. On the other hand, in the combination of TACE and systemic therapy in the TACTICS-L trial [60], the combination of TACE + lenvatinib showed no difference in efficacy within/outside the U-T-7 and within/outside the Milan criteria. It showed a good antitumor effect regardless of the tumor burden. It has also been reported to be effective regardless of the gross tumor type.

### 2.5. Early Stage

HCC poses a considerable risk of recurrence despite radical interventions such as liver resection or RFA. Recurrence typically manifests in two forms: metastatic recurrence originating from curatively treated tumors, and multicentric carcinogenesis stemming from the background liver disease. While addressing the background liver disease (for example, with antiviral therapy) has probably shown some efficacy in suppressing the latter form of recurrence [61,62,63], our ability to effectively prevent recurrence from the former remains largely unaddressed. Antitumor drugs might help in the prevention of metastatic recurrence originating from curatively treated tumors, which decreases the risk of recurrence and could be expected to prolong survival.

Table 4 presents the clinical trials of adjuvant therapy. Until recently, pharmacological interventions capable of preventing metastatic recurrence were yet to be identified [64,65]. However, the IMbrave050 study recently revealed that ATZ/BEV therapy could significantly extend recurrence-free survival (RFS) among individuals deemed to be at high risk of recurrence, as evidenced by an HR of 0.72 (95% CI: 0.53–0.98, *p*-value = 0.012) [66]. One critical consideration is whether this extension in RFS translates into a corresponding prolongation in OS. However, the OS data are presently deemed immature, necessitating future updates to provide additional insights.

The prognosis and available treatment options following HCC relapse after radical treatment vary substantially depending on the nature of the recurrence. Extrahepatic metastatic recurrence or multiple intrahepatic recurrences can present challenges for radical treatment. Conversely, when faced with a single small nodule or intrahepatic recurrence of three or fewer nodules, each measuring ≤ 3 cm, repeated radical interventions, such as liver resection or RFA, are feasible. This unique treatment approach, specific to HCC, distinguishes it from other cancers and is one of the most important points to deliberate when considering adjuvant chemotherapy. On the other hand, the IMbrave050 trial reported that grade 3 or 4 AEs occurred in 41% of patients treated with ATZ/BEV, while 10% experienced grade 3 or 4 irAEs, 8% warranted systemic corticosteroid therapy. IrAEs, which in some cases require permanent treatment, also seem to be a very important issue to consider for adjuvant chemotherapy. Thus, rather than applying adjuvant chemotherapy universally to all cases at high risk of recurrence, it may be more beneficial to target populations facing non-curable recurrence—instances where curative treatment is challenging owing to the tumor condition upon recurrence. It is also crucial to delineate the risk factors associated with such situations. Given that recurrence exceeding the Milan criteria may complicate radical care [67], identifying risk factors for such recurrence holds substantial clinical relevance. However, with the advancements in systemic therapy, particularly in intermediate-stage cases, as mentioned earlier, there is a rising trend of achieving cancer-free and drug-free status. Hence, it is imperative to ascertain which patients can derive true benefits from adjuvant chemotherapy in the future.

Other ongoing phase 3 trials include CheckMate 9DX (NCT03383458), investigating nivolumab versus placebo; EMERALD-2 (NCT03847428), evaluating durvalumab with or without bevacizumab versus placebo; and KEYNOTE-937 (NCT03867084), comparing pembrolizumab against placebo. The outcomes of these trials are eagerly anticipated.

## 3. Combination and Collaboration of Systemic and Locoregional Therapies

### 3.1. Combined Therapy with Systemic Therapy and TACE

#### 3.1.1. Intermediate Stage

To date, most phase 3 trials of TACE in combination with systemic therapy for intermediate HCC have failed to readily demonstrate clinical efficacy [68,69,70,71] (Table 5). The TACTICS trial, a phase 2 study, also failed to demonstrate any significant improvement in OS (mOS: TACE + sorafenib: 36.2 months [95% CI: 30.5–44.1] and TACE alone: 30.8 months [95% CI: 23.5–40.8]; HR 0.861 [95% CI: 0.607–1.223], *p* = 0.40) [72,73]. However, sorafenib plus TACE significantly prolonged PFS when compared with TACE alone (mPFS: TACE + sorafenib: 22.8 months [95% CI: 18.4–27.5] and TACE alone: 13.5 months [95% CI: 9.2–20.6]; HR; 0.661 [95% CI: 0.466–1.938], *p* = 0.02). Using the Response Evaluation Criteria in Cancer of the Liver (RECICL), the ORR/DCR were 28.8/71.3% in the TACE + sorafenib group and 27.6/61.8% in the TACE-alone group, respectively, with no significant difference noted between the two groups. Although the difference in OS between sorafenib plus TACE and TACE alone was 5.4 months [73], it did not reach statistical significance; however, it was deemed to be clinically meaningful. The intermediate stage encompasses a highly diverse population, including cases with relatively favorable prognoses, where the introduction of post-treatment may be relatively easy, even if TACE was ineffective. Consequently, the advantage observed in PFS may not necessarily translate into a significant improvement in OS. Hence, the use of OS as an endpoint in trials targeting the intermediate stages needs to be reconsidered. The success of the TACTICS trial may be attributed to the fact that, unlike the phase 3 trial discussed above, it did not consider new lesions as PD, as it used the RECICL criteria rather than the RECIST criteria. As a result, the fact that the duration of sorafenib administration was maintained to a certain extent may have contributed to the results. If the existing lesions are controlled to some extent, even if a few new lesions appear, the TACE-based treatment strategy may not be doomed to fail, given the nature of locoregional therapy, which only treats visible lesions.

TACTICS-L, a single-arm phase 2 study, combined TACE with lenvatinib, a drug known for its superior ORR when compared with that of sorafenib, and the results have been published [60]. The PFS was 28.0 months (90% CI: 25.1–31.0), with CR rate/ORR among best responses of 67.7%/66.1% and 88.7%/85.5% for RECICL/mRECIST evaluation, respectively. Furthermore, investigations by Kuroda et al. [74], Fu et al. [75], and others [76] have reported promising outcomes upon combining lenvatinib and TACE. Although the methods of combining TACE and lenvatinib differ, good results may be expected with these combinations. The patient demographics enrolled in the TACTICS and TACTICS-L trials share similarities, although direct comparisons, in principle, cannot be made. Nonetheless, the outcomes observed in the TACTICS-L trial notably surpassed those in the TACE + sorafenib group, indicating promising expectations in terms of the OS results. The rationale underlying the combination of TACE and VEGF drugs, as seen in the TACTICS and TACTICS-L trials, can be explained by several factors: (1) potentiation of TACE efficacy via the normalization of tumor blood vessels and reduction in tumor interstitial pressure, (2) inhibition of tumor progression by blocking VEGF system signals released after TACE, and (3) constriction of embolization range due to tumor size reduction from that prior to drug administration, thus possibly preserving liver function [77,78]. This treatment strategy appears highly reasonable for addressing TACE’s limitations and potentially alleviating the burden on liver function.

**Table 5:** Clinical trials of combination therapy comprising systemic therapy and TACE in intermediate-stage HCC.

Study Name	Phase	Site	Regimen	Primary Endpoint	Key Results
Japan-Korea Post-TACE [68]	3	Japan, Korea	TACE + Sorafenib vs. TACE	TTP ^♭^	TTP; sorafenib/placebo: 5.4 [95% CI, 3.8–7.2] months/3.7 [95% CI, 3.5–4.0 months], HR 0.87 [95% CI, 0.70–1.09], *p* = 0.252
SPACE [69]	3	US, Europe Asia–Pacific	DEB-TACE + Sorafenib vs. DEB-TACE	TTP (mRECIST)	TTP; sorafenib plus DEB-TACE/DEB-TACE: 169 [95% CI, 166–219] days/166 [95% CI, 113–168] days, HR 0.797 [95% CI, 0.588–1.080], one sided *p* = 0.072
BRISK-TA [70]	3	Global	TACE + Brivanib vs. TACE	OS	OS; TACE + Brivanib/TACE: 26.4 [95% CI, 19.1-not reached] months/26.1 [95% CI, 19.0–30.9] months, HR 0.90 [95% CI, 0.66–1.23], *p* = 0.5280
ORIENTAL [71]	3	Japan, Korea, Taiwan	TACE + Orantinib vs. TACE	OS	OS; 31.1 [95% CI 26.5–34.5) months/32.3 [95% CI, 28.4–not reached] months, HR 1.090 [95% CI 0.878–1.352] *p* = 0.435
EMERALD-1 [79]	3	Global	TACE + Durvalumab ± Bevacizumab vs. TACE	PFS (RECIST v1.1); TACE + Durvalumab + Bevacizumab vs. TACE	PFS; durvalumab + bevacizumab + TACE/TACE: 15.0 [95% CI 11.1–18.9] months/8.2 [95% CI 6.9–11.1] months, HR 0.77 [95% CI 0.61–0.98], *p* = 0.032
LEAP-012 [80]	3	Global	TACE + Lenvatinib + Pembrolizumab vs. TACE	PFS (RECIST v1.1)	Unpublished
TALENTACE (NCT04712643)	3	China, Japan	TACE + Atezolizumab + Bevacizumab vs. TACE	TACE PFS *	Unpublished
TACE-3 (NCT04268888)	3	UK	TACE + Nivolumab vs. TACE	OS	Recruiting
CA209-74W (NCT04340193)	3	Global	TACE + Nivolumab + Ipilimumab vs. TACE + Nivolumab vs. TACE	Safety and tolerability	Unpublished
TACTICS [73]	2	Japan	TACE + Sorafenib vs. TACE	OS, PFS ^#^ (RECICL)	OS; TACE + sorafenib/TACE: 36.2/30.8 HR 0.861 (95% CI 0.607–1.223), *p* = 0.40, PFS; TACE + sorafenib/TACE: 22.8/13.5 HR0.661 (95% CI 0.466–0.938), *p* = 0.02
TACTICS-L [60]	2	Japan	TACE + Lenvatinib	PFS ^#^ (RECICL)	PFS; 28.0 (90% CI 25.1–31.0), OS not reached

TACE, transcatheter arterial chemoembolization; DEB, drug-eluting bead; HAIC, hepatic arterial infusion chemotherapy; TTP, time to progression; PFS, progression-free survival; OS, overall survival; RECICL, Response Evaluation Criteria in Cancer of the Liver; HR, hazard ratio; CI, confidence interval. TTP ^♭^ is defined as the time to recurrence in patients with CR and TTP in those with non-CR upon study entry. Progression was defined as a ≥25% increase in tumor size or development of a new lesion. TACE PFS * is defined as the time from randomization to untreatable progression, TACE failure/refractoriness, or any cause of death, whichever occurs first, as determined by the investigator (INV). PFS ^#^ is the time from enrollment to untreatable progression (TTUP) or death. TTUP is defined as follows: time to transient liver function deterioration to Child–Pugh C, time to intrahepatic tumor progression (>25% compared with the baseline tumor burden), time to vascular invasion, time to extrahepatic spread, or time to TACE failure.

Advances in combination therapy involving TACE and systemic therapy for intermediate-stage HCC are being actively explored, with numerous phase 3 trials currently underway. As summarized in Table 4, the majority of these regimens involve combining TACE with ICIs, and regimens that combine them with anti-VEGF inhibitors are also being developed.

The results of EMERALD-1, presented at the 2024 ASCO-GI conference, revealed that the mPFS (95%CI) was significantly longer in the TACE + durvalumab + bevacizumab group (15.0 [11.1–18.9] months) than in the TACE-alone group (8.2 [6.9–11.1] months), with an HR of 0.77 (0.61–0.98) and a *p*-value of 0.032 [79]. This study highlights the achievement of the primary endpoint in intermediate-stage HCC. Although these positive findings from a phase 3 trial are promising for the treatment of HCC, the disclosure of OS results in the future will provide further clarity regarding the clinical relevance of these findings.

Other notable phase 3 studies include the LEAP012 trial, evaluating PFS based on the RECIST v1.1 criteria and OS in patients receiving TACE + lenvatinib + pembrolizumab compared with those receiving TACE alone [80]. Additionally, the TALENTACE study (NCT04712643) has examined the PFS and OS outcomes in patients treated with TACE + ATZ/BEV versus TACE alone. Furthermore, the ongoing TACE-3 trial (NCT04268888) comprises both phase 2 and phase 3 components, examining the time to TACE progression and OS in patients receiving TACE + nivolumab versus TACE alone. The CheckMate-74W study (NCT04340193) has evaluated the safety and tolerability of nivolumab (with and without ipilimumab) in combination with TACE, compared to TACE alone, in patients with intermediate-stage HCC.

#### 3.1.2. Advanced Stage

The LAUNCH trial demonstrated the profound impact of adding TACE to treat intrahepatic lesions, even in advanced stages [81]. In patients with advanced HCC, combining lenvatinib with TACE substantially improved both OS and PFS when compared with lenvatinib monotherapy. Importantly, this improved efficacy was observed in cases with extrahepatic metastasis, as well as in several instances of vascular invasion. Consequently, there is potential for developing treatment strategies that integrate TACE with systemic therapy in advanced-stage HCC. Currently, the preferred initial systemic therapies include ATZ/BEV or DUR/TRE. Future studies need to determine whether these drug treatments remain effective when co-administered with TACE.

### 3.2. Combined Therapy with Systemic Therapy and Hepatic Arterial Infusion Chemotherapy (HAIC)

Systemic therapy in combination with HAIC has also been explored (Table 6). The SILIUS trial [82] conducted by Kudo et al. did not detect any survival benefit by adding low-dose cisplatin and fluorouracil HAIC to sorafenib using a port-catheter system, although a sub-analysis suggested efficacy in HCC patients with Vp4. Conversely, He et al. [83] showed a survival benefit upon combining FOLFOX HAIC with sorafenib using a port-catheter system in HCC patients with portal vein invasion. Ikeda et al. [84] also revealed the efficacy of combining cisplatin HAIC with sorafenib therapy for HCC using Seldinger’s method, albeit in a phase 2 trial. Furthermore, a phase 1/2 trial, known as the LEOPARD [85] trial, has recently examined the potential of combining lenvatinib with HAIC. Despite the small number of cases, the study reported favorable response rates, PFS, and OS, along with good tolerability in terms of safety. Further therapeutic developments can be anticipated through phase 3 trials and combining HAIC with immunotherapy.

## 4. New Treatment Concepts and Challenges

Recently, there have been reports indicating favorable outcomes when systemic therapy facilitates successful conversion to resection, RFA, or TACE. Kudo et al. conducted a retrospective analysis of 110 consecutive cases where ATZ/BEV was administered to patients with TACE-unsuitable intermediate-stage HCC [86]. Primarily for cases where partial response (PR) was achieved, local therapy (such as hepatectomy, RFA, and TACE) was added during the disease course, resulting in CR in 35 patients. Considering the three patients where CR was achieved with ATZ/BEV alone, a total of 38 cases (34.5%) attained CR, with 25 of these achieving drug-free status. With advancements in systemic therapy, treatment regimens with high response rates have emerged, emphasizing the importance of seizing conversion opportunities in the future. Traditionally, completely curing intermediate-stage HCC with a high tumor burden has been challenging, with disease control being the primary treatment goal. However, approximately 30% of cases achieve cancer-free status, underscoring the importance of developing treatment strategies that aim for cancer-free outcomes, aided by the progress in systemic therapy and the availability of potent locoregional therapies, which are unique treatments for HCC.

As mentioned above, the recent addition of TACE to lenvatinib therapy to control intrahepatic lesions was found to significantly improve results in terms of PFS/OS [81]. Accordingly, even in cases of advanced-stage HCC, adding locoregional therapy to control intrahepatic lesions could emerge as a viable treatment strategy. Approximately 80% of patients with advanced HCC with extrahepatic lesions face a prognosis of liver-failure-related mortality [87], and studies have indicated that the response of intrahepatic lesions to treatment independently influences prognosis [88,89], further supporting the findings of the LAUNCH trial. In this context, an intriguing phase 3 trial, the IMPACT trial (Table 7), is currently underway, with an overview presented by Yamashita et al. at ESMO Asia 2023 [90]. Importantly, this trial has adopted a unique approach, where the response to ATZ/BEV treatment determines the subsequent treatment scheme. Specifically, patients who have achieved CR or PR at the 12-week imaging evaluation are assigned to the ABC conversion cohort, whereas those with SD are assigned to the SD randomization cohort. Patients who achieve SD are then randomly allocated to either continue ATZ/BEV alone or ATZ/BEV plus intrahepatic control TACE. The conversion rate will be prospectively analyzed in the ABC conversion cohort, while the OS of the two groups will be compared in the SD randomized cohort. The survival curve based on the best response from the IMbrave150 trial revealed that the prognosis of the SD group was significantly poorer than that of the CR/PR group [91]. Hence, a key clinical challenge is to improve outcomes in the SD group. The addition of TACE to SD cases could induce tumor antigen release, activate antigen-presenting cells and, consequently, promote T-cell priming [92], potentially synergizing with ATZ/BEV treatment. It will be interesting to observe whether the efficacy of lenvatinib presented in the LAUNCH trial can also be replicated with ATZ/BEV. Depending on the outcomes of the IMPACT trial, novel response-guided therapeutic strategies may emerge, eliciting exciting advancements in HCC management.

In a phase 2 trial (LEN-HCC; Table 7), Ichida et al. examined the potential for conversion to resection in patients with oncologically or technically unresectable HCC who received lenvatinib, reporting promising results despite the limited number of cases [93]. Given the high recurrence rate of HCC, the emergence of drugs boasting high response rates holds promise for conversion therapy and for the development of neoadjuvant chemotherapy (NAC). Resectability criteria may vary among healthcare facilities, and a precise definition is yet to be established. Thus, in the past, the distinction between conversion therapy and NAC has been slightly ambiguous; however, recent efforts have led to the establishment of clearer definitions of resectability [94]. Henceforth, discussions regarding NAC and conversion therapy are expected to be informed by these refined definitions, facilitating more precise treatment strategies. In an attempt to address NAC, a phase 2 trial of HAIC with cisplatin and lenvatinib combination therapy (LEOPARD-NEO; jRCTs031230128: Table 7) has been initiated for patients with borderline resectable HCC, which is firmly defined beforehand.

Currently, trials are exploring the combination of systemic therapy with HAIC. Although the results may vary between different regimens, HAIC has generally demonstrated clinical efficacy, particularly in HCC cases with vascular invasion [95]. Several studies have reported the efficacy of HAIC while comparing HAIC and sorafenib; however, the emergence of lenvatinib, boasting a high ORR, and combination immunotherapy that exhibits superiority over sorafenib would lead to changes in the interpretation of these results. As mentioned above, Ikeda et al. achieved a remarkably high ORR by combining lenvatinib with cisplatin HAIC, reporting particularly favorable outcomes, even in cases with vascular invasion (LEOPARD trial [85]). HAIC, employing the Seldinger technique, which does not necessitate an implanted port system, offers a straightforward and standardized approach, with verification through phase 3 trials expected in the future.

**Table 7:** New treatment concepts and challenges.

Study Name	Phase	Site	Regimen	Subject	Primary Endpoint	Key Results
IMPACT [90]	3	Japan	Atezolizumab + Bevacizumab	Unresectable, excluding Vp3/Vp4	SD randomized cohort; OS ABC conversion cohort; conversion rate	Recruiting
LENS-HCC [93]	2	Japan	Lenvatinib	Oncologically or technically unresectable	Surgical resection rate	Resection rate: oncologically unresectable; 76.2% technically unresectable; 14.3%
LEOPARD-NEO (jRCTs031230128)	2	Japan	Lenvatinib + HAIC with Cisplatin	Borderline resectable	Resection rate	Recruiting
Case reports [96]			Immunotherapy	As a neoadjuvant treatment before liver transplantation		There were cases of good outcome, cases of re-transplantation, and a case of fatal hepatic necrosis

Vp3, tumor thrombus involving the first branches of the portal vein; Vp4, tumor thrombus involving the main trunk of the portal vein; SD, stable disease; OS, overall survival; ABC conversion, atezolizumab–bevacizumab curative conversion.

Recently, scattered reports have addressed the use of immunotherapy as a neoadjuvant treatment before liver transplantation [96] (Table 7). In particular, immunotherapy is complicated by various clinical challenges, such as rejection, which should be clarified and overcome in the future.

Thus, advances in systemic therapy could lead to treatment courses that have rarely been employed in the clinical practice of HCC in the past, and advances in systemic therapy are constantly changing treatment concepts and strategies.

## 5. Current Status and Future Perspectives

Systemic therapy for HCC has undergone rapid advancements, with numerous phase 3 trials, including both drug combination and drug combination with locoregional therapy, underway to further enhance treatment outcomes. In clinical practice, it is markedly crucial to maximize the efficacy of existing drugs while minimizing AEs. In this regard, the development of biomarkers that are capable of predicting both efficacy and AEs would be extremely valuable. The emergence of drugs with high ORRs has revolutionized the treatment landscape, prompting the initiation of new trials. Both in clinical practice and in the development of novel treatments, a deep understanding of the clinical characteristics of HCC, the strengths and limitations of available treatment modalities, and the efficacy and safety profiles of each drug is crucial for optimal patient management.

## Figures and Tables

**Table 1:** Phase 3 trials of first-line treatment.

Study Name	Phase	Site	Regimen	Primary Endpoint	Key Results
COSMIC-312 [32]	3	Global	Cabozantinib + Atezolizumab vs. Sorafenib	PFS (RECIST v1.1), OS	PFS; cabozantinib + atezolizumab/sorafenib: 6.8 [99% CI 5.6–8.3]/4.2 [2.8–7.0] months, HR 0.63 [99% CI 0.44–0.91], *p* = 0.0012 mOS; cabozantinib + atezolizumab/sorafenib 15.4 [96% CI 13.7–17.7]/15.5 [12.1–not estimable] months, HR 0.90 [96% CI 0.69–1.18], *p* = 0.44
CheckMate 9DW (NCT04039607)	3	Global	Nivolumab + Ipilimumab vs. Sorafenib or Lenvatinib	OS	mOS; 23.7 months for nivolumab plus ipilimumab vs. 20.6 months for lenvatinib or sorafenib (HR, 0.79; 95% CI, 0.65–0.96; *p* = 0.0180) ORR; nivolumab plus ipilimumab; 36% vs. 13% for lenvatinib or sorafenib (*p* < 0.0001). Duration of response of 30.4 months for nivolumab plus ipilimumab compared to 12.9 months for lenvatinib or sorafenib
LEAP-002 [33]	3	Global	Lenvatinib + Pembrolizumab vs. Lenvatinib	PFS (RECIST v1.1), OS	PFS; lenvatinib + pembrolizumab/lenvatinib: 8.2 [95% CI 6.4.–8.4]/8.0 [95% CI 6.3–8.2] months, HR 0.87 [95% CI 0.73–1.02], stratified log-rank *p* = 0.047 mOS; lenvatinib + pembrolizumab/lenvatinib: 21.2 [95% CI 19.0–23.6]/19.0 [95% CI 17.2–21.7] months, HR 0.84 [95% CI 0.71–1.00] stratified log-rank *p* = 0.023 Lenvatinib plus pembrolizumab did not meet pre-specified significance for improved OS and PFS compared to lenvatinib alone
IMbrave152 (NCT05904886)	3	Global	Atezolizumab + Bevacizumab + Tiragolumab vs. Atezolizumab + Bevacizumab	PFS (RECIST v1.1), OS	Recruiting
SIERRA * (NCT05883644)	3	US, Europe, Asia	Duruvalumab + Tremelimumab	Incidence of grade 3 or 4 possibly related to treatment adverse events (PRAEs), ORR	Recruiting

PFS, progression-free survival; OS, overall survival; mOS, median OS; ORR, objective response rate. The subjects in SIERRA* study were recruited as follows: (a) C-P A with PS2, (b) C-P 7/8 with PS0/1, (c) C-P A with PS of 0/1 and with evidence of chronic main trunk portal vein thrombosis at enrolment.

**Table 2:** Gr3 or higher adverse events that occcurred in 3% or more of all patients for each drug/regimen reported in clinical trials.

	Sorafenib (%) [1]	Lenvatinib (%) [3]	Atezolizumab + Bevacizumab (%) [6]	Durvalumab + Tremelimumab (%) [7]	Durvalumab (%) [7]
Hypertension		23 (14)	15.2 (12.2)		
Proteinuria		6 (2)	3 (0.6)		
Diarrhea	8	4 (4)		4.4 (4.3)	
Aspartate aminotransferase increased		5 (8)	7 (5.1)	5.2 (3.2)	6.7 (3.2)
Alanine aminotransferase increased			3.6 (1.3)		3.1 (1.9)
Amylase increased				3.6 (1.1)	
Lipase increased				6.2 (2.9)	4.1 (2.9)
Hyponatremia				4.1 (2.9)	
Platelet count decrease		5 (3)	3.3 (1.3)		
Palmar–plantar erythrodysesthesia	8	3 (11)			
Decreased appetite		5 (1)			
Decreased weight		8 (3)			
Fatigue	3	4 (4)			
Increased blood bilirubin		7 (5)			
Discontinuation rate due to AEs	11 ^#^	9 (7) ^#^	7 (10.3) ^#^	13.7 (16.8) ^#^	8.2 (16.8) ^#^
Systemic steroid use rate due to irAEs/imAEs	1.9 *		12.2	20.1 *	9.5 *

irAE, immune-related adverse event, imAE, immune-mediated adverse event. Data represent the results in each pivotal study. Numbers in parentheses show the results for sorafenib, which was the control arm; * shows the percentage of high-dose glucocorticoid use in the HIMALAYA trial [7]. ^#^ indicates discontinuation rate due to treatment-related AEs.

**Table 3:** Clinical trials in intermediate-stage HCC.

Study Name	Phase	Site	Regimen	Primary Endpoint	Key Results	Remarks
REPLACEMENT [55]	2	Japan	Atezolizumab + Bevacizumab	PFS (mRECIST)	Median PFS 9.1 [95% CI: 7.1–10.2] months	Comparison with TACE historical data using PSM; PFS
ABC-HCC (NCT04803994)	3	Europe, Japan, Korea	TACE vs. Atezolizumab + Bevacizumab	Time to failure of treatment strategy *	Recruiting	

TACE, transcatheter arterial chemoembolization; PFS, progression-free survival; PSM, propensity score matching. “Failure of treatment strategy *” is reached in case of progressive disease accompanied by any of the following: loss of clinical benefit, unacceptable toxicity, liver function deterioration, therapy not further applicable for other reasons.

**Table 4:** Clinical trials of adjuvant therapy.

Study Name	Phase	Site	Regimen	Primary Endpoint	Key Results
C000000445 * [64]	3	Japan	UFT vs. Placebo	RFS	RFS at 5 year; UFT/placebo: 29%/29% HR 1.01 [95% CI: 0.84–1.22], *p* = 0.87
STROM [65]	3	Global	Sorafenib vs. Placebo	RFS	RFS; Sorafenib/placebo: 33.3/33.7 months, HR 0.940 [95% CI 0.780–1.134], one-sided *p* = 0.26
IMbrave050 [66]	3	Global	ATZ/BEV vs. Placebo	RFS	RFS; ATZ/BEV/placebo NE (not evaluable) [95% CI 22.1-NE]/NE [95% CI 21.4-NE], HR 0.72 [adjusted 95% CI 0.53–0.98], *p* = 0.012
CheckMate 9DX (NCT03383458)	3	Global	Nivolumab vs. Placebo	RFS	Unpublished
EMERALD-2 (NCT03847428)	3	Global	Durvalumab ± Bevacizumab vs. Placebo	RFS (Durvalumab + bevacizumab vs. placebo)	Unpublished
KEYNOTE-937 (NCT03867084)	3	Global	Pembrolizumab vs. Placebo	RFS	Unpublished

RFS, recurrence-free survival; CI, confidence interval. * Registration number: C000000445, accessible in the Clinical Trials Registry managed by the University Hospital Medical Information Network in Japan, which can be accessed free on the internet (www.umin.ac.jp/ctr/index.htm, accessed on 5 April 2016).

**Table 6:** Clinical trials of combination therapy comprising systemic therapy and HAIC.

Study Name	Phase	Site	Regimen	Primary Endpoint	Key Results
SILIUS [82]	3	Japan	Sorafenib + HAIC (LFP) vs. Sorafenib	OS	OS; sorafenib + LFP/sorafenib; 11.8 [95% CI, 9.1–14.5] months/11.5 [95% CI, 8.2–14.8] months, HR 1.009 [95% CI, 0.743–1.371], *p* = 0.955 Vp4: OS; sorafenib + LFP/sorafenib; 11.4 [95% CI, 7.0–15.9] months/6.5 [95% CI, 4.5–8.4] months, HR 0.493 [95% CI, 0.240–1.014], *p* = 0.050
NCT02774187 [83]	3	China	Sorafenib + HAIC (FOLFOX) vs. Sorafenib	OS	OS; sorafenib + FOLFOX/sorafenib: 13.37 [95% CI, 10.27–16.46] months/7.13 [95% CI, 6.28–7.98] months, HR 0.35 [95% CI, 0.26–0.48], *p* < 0.001
UMIN000005703 [84]	2	Japan	Sorafenib + HAIC (Cisplatin) vs. Sorafenib	OS stratified by the allocation factors, including the presence/absence of portal vein tumor thrombosis and extrahepatic metastases	OS; sorafenib + cisplatin/sorafenib: 10.6 months/8.7 months, stratified HR 0.60 [95% CI, 0.38–0.96], *p* = 0.031
LEOPARD [85]	2	Japan	Lenvatinib + HAIC with Cisplatin	Objective response rate (central judgment: modified RECIST)	ORR; 64.7 [95% CI, 46.5–80.3]%, DCR; 76.5 [95% CI, 58.8–89.3]%

UMIN-CTR (http://www.umin.ac.jp/ctr/index-j.htm, accessed on 5 April 2016); LFP, low-dose cisplatin and fluorouracil; FOLFOX, oxaliplatin, fluorouracil, and leucovorin; OS, overall survival; HR, hazard ratio; CI, confidence interval; ORR, objective response rate; DCR, disease control rate; HAIC, hepatic arterial infusion chemotherapy.
